# Supplementary material for: Mothers’ and Grandmothers’ misconceptions and socio-cultural factors as barriers to exclusive breastfeeding: A qualitative study involving Health Workers in two rural districts of Ghana
Source: PLoS One. 2020 Sep 17;15(9):e0239278. doi: 10.1371/journal.pone.0239278 (PMC7498105; doi:10.1371/journal.pone.0239278)
Supplement: Attachment — Submitted filename: Reviewer report_EBF in Ghana_Doris.pdf [file pone.0239278.s001.pdf]

Reviewer report

Title: Mothers' misconceptions and socio-cultural factors prevent exclusive breastfeeding:

findings from two rural districts in Ghana

Authors: Nsiah-Asamoah et al

COMMENTS

General comments

- It is a good article, highlighting misconceptions and cultural barriers and shows what the HWs know about this in relation to the mothers they deal with. It is important for HWs to understand the community beliefs so that they can appropriately deal with them, beyond only tackling the clinical interventions.

- Authors need to read through and make grammatical edits
  - E.g. sentence 1 under Conclusions section in the abstract
  - Sentence 1 of introduction is long and could be better edited or divided into 2, and other grammatical errors in document.

Major Compulsory Revisions

Overall methods comment

Using qualitative guidelines for paper writing will help to ensure that the methods are more fully described e.g. COREQ or other guidelines. Authors would be able to add more information on things like participant refusals or drop outs, places where interviews were held, a brief indication of what was in the FGD guides, training of research assistants, research team and reflexivity, among others.

Data analysis

- More details also needed for the analysis, for instance which kind of coding, did the coding team hold discussions to come up with themes, who did the coding, was analysis done manually or using software, was data saturation discussed, etc.

Results

Under section about grandmothers not doing EBF

- The 1st quote seems to be what HWs heard from the grandmothers, but not what the mothers themselves perceived.
- The last paragraph on that page (page 5/11) belongs to the discussion section because it is going beyond reporting results to listing their implications. The same applies to the first paragraph on page 7 (13), as well as later on the same page where authors discuss feeding of male babies, later on page 8 and in other places in the results – page 9 on herbal concoctions even cites other literature.

Limitations

- While it is good to understand the HWs view points, they are providing “second hand” information from the mothers, so it is not directly from the mothers, which is a limitation of this study.
- I think what is also missing was verification of whether HWs actually believe these things too, especially if they are from the same community. This could affect their delivery of health education.

Conclusion

- Good recommendations made. However, the difficulty in changing cultural beliefs needs to be acknowledged, and the need for innovation therein and possibly borrowing from other behavioural change interventions around culture.

Minor Essential Revisions

Introduction

- The literature and examples of barriers to EBF in the introduction only focus on cultural issues and misconceptions. There are other barriers to EBF and it would be good to briefly mention these as well.
- The last paragraph of the introduction explains why the CHWs and CHVs need to know the myths. Authors need to explain why they focused on these 2 groups only and maybe not the other HWs who deal with mothers and may be key to initiating breastfeeding at birth, for instance midwives.
- Also when authors refer to HWs, do they specifically mean only the CHWs and CHVs for this study? This needs to be clear to avoid confusion.

Study design and population

- Paragraph 1, last sentence: please specify which group of people you refer to when you say underweight. Is it children, babies, etc?
- Paragraph 2: Why should the HWs have been working for at least 5 years in the district?
- More information may be needed on the random sampling process, and how many were from CWCs and from the communities? Are these CWCs in hospitals?
- Do we have the demographics of the HWs?
- How were participants recruited? E.g. face to face, etc?

Data Collection

- Sentence 1: Focus not Focused

Data analysis

- Thematic analysis and thematic framework analysis are being used interchangeably, would you like to pick 1?

Results

- There is some repetition in the results section, for example when authors introduce the theme and then go on to the sub-themes, they need to reduce repetition of words there.

Discussion

- Remember to mention in which country or region the studies you are citing were done and possibly any limitations or strengths of the study – critique some of them.
- Highlight study strengths

Conclusion

- It may be better to have the recommendations well outlined in the discussion rather than in the conclusion.

Discretionary Revisions

Introduction

- Is there another source of breastfeeding data in Ghana beyond the GDHS? It would be a good addition to the literature, to back up the statements. This is because the DHS also has some reporting challenges. For instance, is EBF reducing or it could also be issues around reporting and data collection?

Discussion

- It would be good to elaborate on motivational theory so readers don't have to look for it
- For studies on HWs not EBF, what were the reasons for this?
